# Treatment of presumed *Nocardia* endophthalmitis and subretinal abscess with serial intravitreal amikacin injections and pars plana vitrectomy

**DOI:** 10.1186/s12348-020-00205-3

**Published:** 2020-05-27

**Authors:** Sara L. Hojjatie, Sherveen S. Salek, William A. Pearce, Jill R. Wells, Steven Yeh

**Affiliations:** Department of Ophthalmology, Emory Eye Center, Emory University School of Medicine, 1365B Clifton Rd NE, Atlanta, GA 30322 USA

## Abstract

A 64-year-old man with a past medical history of liver transplantation on chronic immunosuppressive therapy presented with gradual worsening of vision over 2 months in his right eye. His recent history of *Aspergillus* and *Nocardia* pneumonia with positive bronchoalveolar lavage, in concert with vitritis and subretinal abscess, were concerning for endogenous endophthalmitis. A sputum culture and transbronchial lung biopsy stains grew *Nocardia farcinica* although aqueous humor sampling was negative. He was treated with four serial amikacin intravitreal injections over the course of 4 weeks. Pars plana vitrectomy for worsening macular traction and subsequent cataract surgery resulted in significant clinical and anatomic improvement of vision to 20/60 and consolidation of the subretinal abscess.

## Introduction

*Nocardia* is a gram-positive, saprophytic, aerobic bacterium that is an opportunistic infection in immunocompromised patients [1]. It is most commonly seen in the lungs, brain, and soft tissues, and rarely seen as an intraocular infection [1]. Ocular infection often has extremely poor outcomes, often due to a low index of suspicion causing delays in diagnosis [2]. Because *Nocardia* is a rare ophthalmic infection, there is sparse literature describing cases of *Nocardia* endophthalmitis. Previous studies have shown a high sensitivity of *Nocardia* to amikacin, which interferes with protein synthesis [2]. One study found that amikacin can be the drug of choice for intravitreal injections due to its favorable susceptibility pattern [3]. Here, we present a case of *Nocardia* endophthalmitis that was treated with serial amikacin injections and pars plana vitrectomy.

## Case report

A 64-year-old man presented with gradual painless decrease of vision and floaters in the right eye for 6 weeks. His history was notable for hepatocellular carcinoma requiring orthotopic liver transplant 2 years prior, and he remained on chronic immunosuppressive therapy including mycophenolate mofetil, tacrolimus, and prednisone. He was hospitalized 3 months prior to presentation for cavitary pneumonia, with bronchoalveolar lavage (BAL) cultures growing *Nocardia farcinica* and *Aspergillus fumigatus*. He was treated with voriconazole and trimethoprim-sulfamethoxazole (Bactrim), which was later switched to ciprofloxacin due to renal toxicity.

**Fig. 1:**
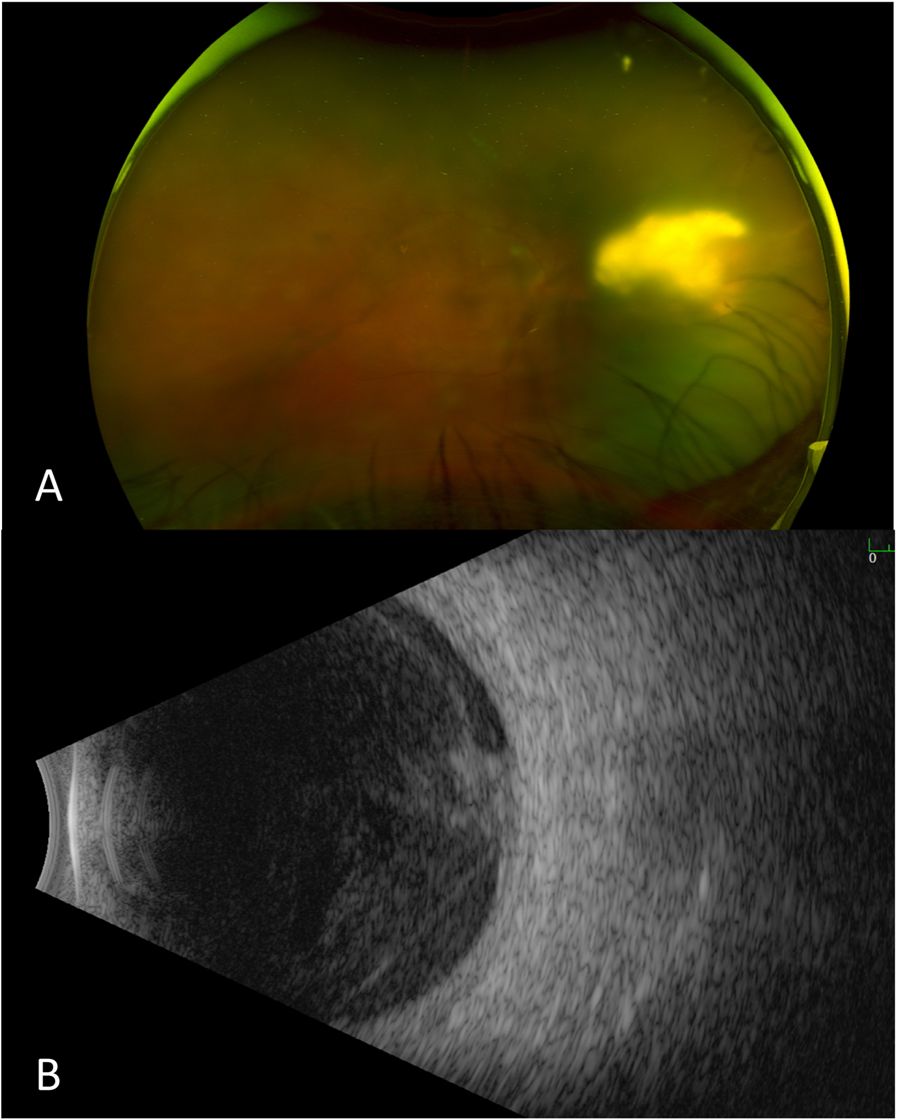
**a** Fundus photo and **b** B-scan showing a vitreous mass with white debris extending inferiorly concerning for endogenous endophthalmitis

The patient appeared well-nourished and in no acute distress. Ophthalmic examination showed a visual acuity (VA) of 20/300 in the right eye and 20/25 in the left eye. Anterior segment exam OD revealed 3+ anterior chamber cell and 3+ vitreous cell and haze. Fundus examination of the right eye showed a yellow-white subretinal mass nasal to the optic nerve and extending to the mid-periphery (Fig. 1).

The differential diagnosis included infectious and autoimmune etiologies, as well as post-transplant, Epstein Barr virus (EBV)-associated lymphoproliferative disorder. The patient underwent a vitreous tap, which was unsuccessful at obtaining a vitreous specimen, followed by aspiration of anterior chamber (AC) fluid. Cultures of AC fluid grew no organisms and no PMNs. He was given an intravitreal injection of voriconazole and vancomycin. Intravitreal ceftazidime was held because the strain was resistant to cephalosporins. Amikacin was initially held for potential aminoglycoside toxicity. The patient was started on topical corticosteroids and cycloplegic eye drops.

The initial BAL tissue specimen and sputum culture later grew *Nocardia farcinia* that was susceptible to Bactrim, ciprofloxacin, linezolid, and amikacin. Due to issues with receiving Bactrim, the patient was given oral voriconazole and levofloxacin, as well as the first dose of intravitreal amikacin.

**Fig. 2:**
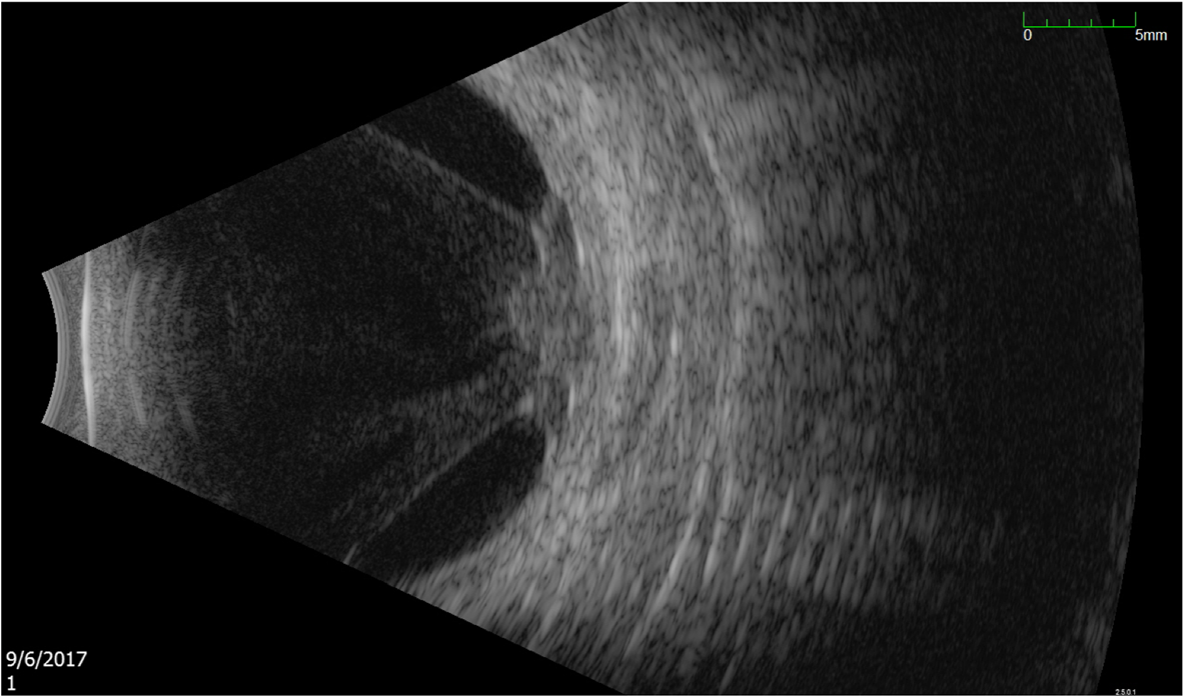
B-scan ultrasound demonstrating worsening nasal retinal traction at follow-up visit

**Fig. 3:**
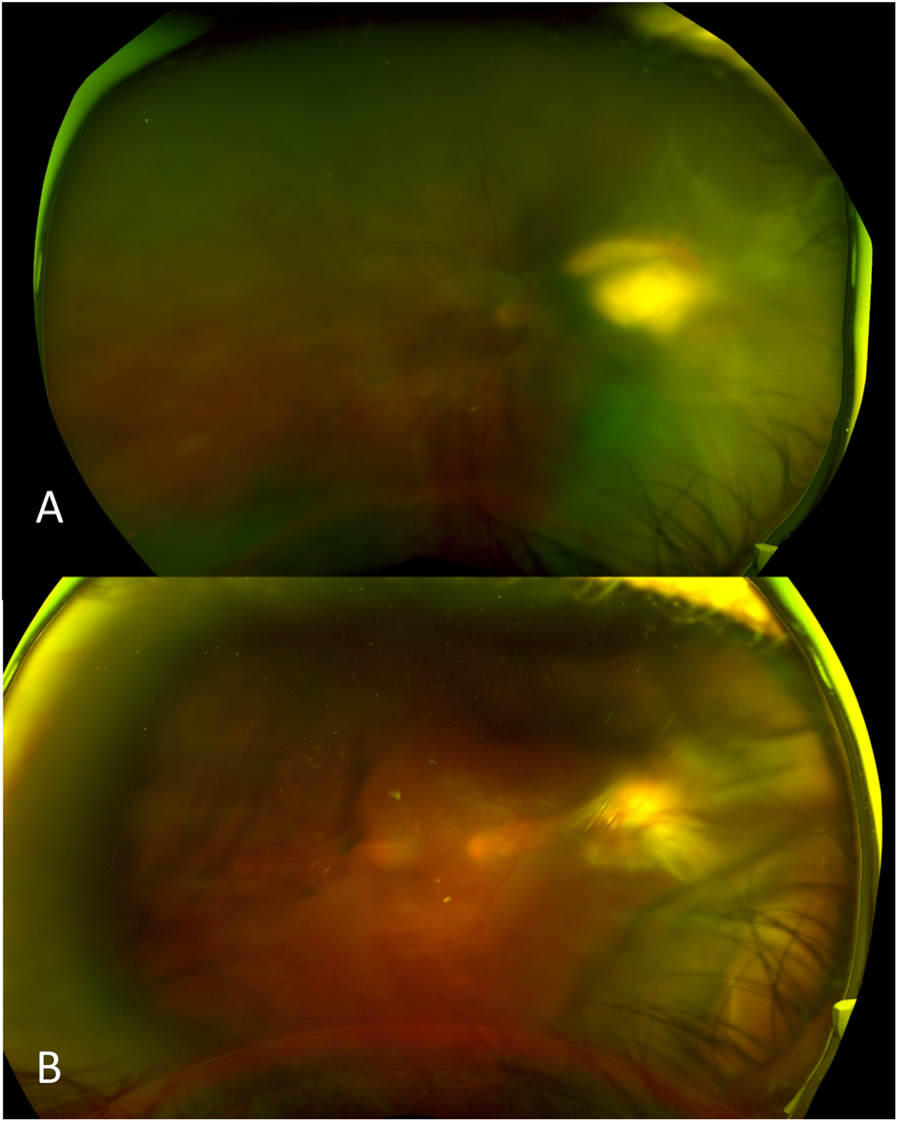
**a** Pre-operative after amikacin injections and **b** post-operative week 2 fundus images

At follow-up, B-scan showed new nasal retinal traction in the right eye (Fig. 2). Given persistent vitritis and active-appearing subretinal lesion, intravitreal amikacin was given again. Over 4 weeks, four doses of 0.2 mg of intravitreal amikacin were administered into the vitreous overlying the abscess. Three months after initial presentation, the patient underwent pars plana vitrectomy and membrane peel. Because the abscess appeared inactive, retinotomy and debridement were deferred, and the dense overlying membrane was removed. Vitreous fluid aspirate from the surgery was negative for bacterial and fungal organisms. At post-operative week two, the patient showed improvement of vision to 20/60 and a decrease in the size of the subretinal abscess (Fig. 3). Cataract extraction with intraocular lens (IOL) was performed 7 months later with VA of 20/60 at post-op day zero. The patient had a VA of 20/100 at final follow-up, almost a year from initial presentation.

## Discussion

This paper reports a case of endogenous *Nocardia* endophthalmitis in a patient with a history of organ transplant on immunosuppressive medication. His only positive cultures came from bronchoalveolar lavage, sputum, and transbronchial biopsy samples. Despite having an unsuccessful vitreous tap and no microbial growth from anterior chamber fluid, serial amikacin injections for presumed *Norcardia* subretinal abscess and pars plana vitrectomy were successful in improving the patient’s disease process and clinical course.

*Nocardia* endophthalmitis is a serious vision-threatening disorder often found at late stages. Endogenous inoculation of *Nocardia* often causes large choroidal lesions and overlying hemorrhage and vitritis, as seen in this patient. Previous documentation of endogenous endophthalmitis concurrent with pulmonary involvement supports the fact that *Nocardia farcinica* presents in these locations [1]. Although there was no positive growth from AC or vitreous cultures, our patient had positive sputum cultures that were sufficient to suggest a likely *Nocardia* etiology for endogenous endophthalmitis. In another study of a patient with a similar diagnosis, vitreous samples were negative but blood cultures were positive for *Nocardia*, suggesting that blood cultures in the setting of other systemic findings were important to diagnosis [4]. Thus, our case further demonstrates the utility of positive cultures from systemic sources to support a diagnosis of infectious endophthalmitis.

Serial intravitreal amikacin injections proved to be successful in the treatment of the vitritis and subretinal abscess in this case of presumed endogenous *Nocardia* endophthalmitis. Due to susceptibility and clinical experience, amikacin is the antibiotic choice for management of ocular *Nocardia* infections [5, 6]. Nonetheless, the reported risk of macular infarction with intravitreal amikacin should be considered prior to administration [7]. One study supports the use of amikacin for ocular *Nocardia* due to a high level of susceptibility (89.7%) [8]. This same study found that *Nocardia* endophthalmitis often requires aggressive medical and surgical management [8]. Surgical intervention was necessary in our patient to address worsening macular traction from the subretinal abscess.

Outcomes of *Nocardia* endophthalmitis are often poor, likely due to delayed presentation and extensive anterior chamber involvement [8]. Eyes with *Nocardia* endophthalmitis more frequently required multiple surgical interventions to achieve control of infection [8]. A previously reported case of *Nocardia* endophthalmitis treated with repeated intravitreal injections of amikacin and cephazolin over a course of 3 months showed transformation of the lesion into a scar [9]. Thus, there may be benefit in multiple intravitreal injections of amikacin for *Nocardia* endophthalmitis. Close follow-up of these patients is essential in order to determine the timing of this therapy.

In summary, we report a case of endogenous *Nocardia* endophthalmitis in the setting of negative ocular cultures, successfully treated with serial amikacin injections and pars plana vitrectomy. As an infection that can have devastating ocular manifestations, consideration of infection by *Nocardia* in immunocompromised and post-transplant patients and consistent treatment can be associated with a favorable clinical outcome.

## Data Availability

Data sharing is not applicable to this article as no datasets were generated or analyzed during the current study.

## Abbreviations

BAL: Bronchoalveolar lavage
AC: Anterior chamber
EBV: Epstein-Barr virus
PMN: Polymorphonuclear neutrophils
VA: Visual acuity
IOL: Intraocular lens
